# Peripheral Monocyte Functions and Activation in Patients with Quiescent Crohn’s Disease

**DOI:** 10.1371/journal.pone.0062761

**Published:** 2013-04-26

**Authors:** David Schwarzmaier, Dirk Foell, Toni Weinhage, Georg Varga, Jan Däbritz

**Affiliations:** 1 Department of Pediatric Rheumatology and Immunology, University Children’s Hospital Münster, Münster, NRW, Germany; 2 Interdisciplinary Center of Clinical Research, University of Münster, Münster, NRW, Germany; 3 The Royal Children’s Hospital Melbourne, Murdoch Children’s Research Institute, Parkville, VIC, Australia; Glaxo Smith Kline, Denmark

## Abstract

Recent developments suggest a causal link between inflammation and impaired bacterial clearance in Crohn’s disease (CD) due to alterations of intestinal macrophages. Studies suggest that excessive inflammation is the consequence of an underlying immunodeficiency rather than the primary cause of CD pathogenesis. We characterized phenotypic and functional features of peripheral blood monocytes of patients with quiescent CD (n = 18) and healthy controls (n = 19) by analyses of cell surface molecule expression, cell adherence, migration, chemotaxis, phagocytosis, oxidative burst, and cytokine expression and secretion with or without lipopolysaccharide (LPS) priming. Peripheral blood monocytes of patients with inactive CD showed normal expression of cell surface molecules (CD14, CD16, CD116), adherence to plastic surfaces, spontaneous migration, chemotaxis towards LTB4, phagocytosis of *E. coli*, and production of reactive oxygen species. Interestingly, peripheral blood monocytes of CD patients secreted higher levels of IL1β (p<.05). Upon LPS priming we found a decreased release of IL10 (p<.05) and higher levels of CCL2 (p<.001) and CCL5 (p<.05). The expression and release of TNFα, IFNγ, IL4, IL6, IL8, IL13, IL17, CXCL9, and CXCL10 were not altered compared to healthy controls. Based on our phenotypic and functional studies, peripheral blood monocytes from CD patients in clinical remission were not impaired compared to healthy controls. Our results highlight that defective innate immune mechanisms in CD seems to play a role in the (inflamed) intestinal mucosa rather than in peripheral blood.

## Introduction

Recent developments in immunology and genetics have consolidated the view of Crohn’s disease (CD) as a form of immunodeficiency and studies continue to highlight defects of the initially involved innate immune system [1]–[4]. Segal *et al.* performed several *in vitro* studies using monocyte-derived macrophages of CD patients in clinical remission to identify a defective mechanism, which could explain the diminished acute inflammatory response in CD patients. They observed that diminished neutrophil recruitment and bacterial clearance result from insufficient macrophage cytokine secretion on bacterial challenge. As a result, secondary macrophage activation may lead to the formation of granulomas and chronic inflammation [5], [6]. Furthermore, it has been suggested that blood monocytes are the exclusive source of macrophages in inflamed intestinal mucosa [7], [8] and that both peripheral monocytes as well as their derivative cells play an important role in the pathophysiology of CD. We were thus interested in the characterization of peripheral blood monocytes in CD, specifically in the underlying constitutive intrinsic alterations of monocyte subpopulations. The aim of the study was to identify possible functional and phenotypic abnormalities of monocytes already at a stage before their recruitment into the intestinal mucosa. We investigated i) the expression of cell surface molecules; ii) functional characteristics including adherence, migration, chemotaxis, oxidative burst, and phagocytosis; and iii) the expression and secretion of chemokines and pro- and anti-inflammatory cytokines. In addition, priming studies were performed using lipopolysaccharide (LPS) in order to simulate the exposure and subsequent activation of monocytes to proinflammatory bacterial products in the intestinal mucosa.

## Materials and Methods

### Ethics Statement

Written informed consent was obtained from all patients. The study was approved by the ethics committee of the University of Münster, Germany (reference number 2009-434-f-S, J.D.).

### Patients and Controls

Patients who met the inclusion criteria were recruited from January 2010 to December 2011 to participate in the study. All patients had definitive diagnoses of CD, which were confirmed by standard diagnostic criteria, with quiescent disease (Harvey Bradshaw Index [HBI] ≤5) [9]. Patients receiving either no medication, a stable maintenance dose of 5­aminosalicylates (2.5 g/d) or a tapering dose of budesonide for the previous 3 months were included. None of the patients had received conventional/systemic corticosteroids, immunosuppressants, anti­tumor necrosis factor, or metronidazole therapy within 3 months of enrolment. Healthy volunteers approximately matched for age and sex were used as controls. No subject was studied more than once in each of the different experiments. Details of included patients and healthy control subjects are provided (Table 1).

**Table 1:** Characteristics of Crohn’s disease patients and healthy controls.

	Crohn‘s disease	Controls
**Number**, n	18	19
**Age**, years (range)	45 (27–59)	30 (23–47)
**Sex**, n (%)		
- female	10 (55%)	9 (47%)
- male	8 (45%)	10 (53%)
**BMI**, kg/m^2^ (range)	23.6 (18.0–38.2)	21.5 (19.4–32.8)
**Duration of disease**, years (range)	7 (0–45)	–
**Location**, n (%)		
- ileal	6 (33%)	–
- colonic	2 (11%)	–
- ileo-colonic	8 (45%)	–
- other	2 (11%)	–
**Harvey-Bradshaw index**, score (range)	1 (0–5)	–
**Medication**, n (%)		
- Budesonide	4 (22%)	0 (0%)
- Mesalazine	4 (22%)	0 (0%)
- None	10 (56%)	19 (100%)

### Isolation of Peripheral Blood Monocytes and Flow Cytometry

Human peripheral blood mononuclear cells (PBMCs) were isolated from patients and healthy controls by Ficoll-Hypaque (Pharmacia, Freiburg, Germany) density gradient centrifugation. Monocytes were further enriched by using the CD16 monocyte isolation kit in combination with CD14 microbeads according to the manufacturer`s instructions (Miltenyi Biotec, Bergisch Gladbach, Germany). In brief, 1×10^8^ PBMCs were incubated with 100 µl FcR blocking reagent together with 100 µl non-monocyte depletion cocktail containing anti-CD15 and anti-CD56 microbeads to magnetically deplete CD16 expressing NK cells and granulocytes. Subsequently, the flow-through was incubated with 100 µl anti-CD16 and 200 µl anti-CD14 microbeads and CD14/CD16 expressing monocytes were positively selected by magnetic separation.

To determine purity of isolated monocytes and to investigate CD14, CD16 and CD116 expression on naïve PBMC monocytes, 5×10^5^ cells of each PBMCs and isolated monocytes, were stained in 50 µl FACS-buffer (PBS +1% FSC) with anti-CD14-FITC (clone 61D3, eBioscience, San Diego, CA, USA), anti-CD116 Pe (clone 4H1, eBioscience, San Diego, CA, USA) and anti-CD16-APC (clone eBioCB16, eBioscience, San Diego, CA, USA) antibodies for 30 min at room temperature in the dark. After washing twice with FACS-buffer, cells were analyzed using a FACSCalibur and CellQuest software (both from Becton Dickinson, Heidelberg, Germany). Purity after isolation was routinely higher than 90% for CD14/CD16 expressing monocytes.

Monocytes were then cultured (1×10^6^ cells/ml) in hydrophobic teflon bags (Heraeus, Hanau, Germany) in McCoy’s 5a medium supplemented with 15% heat-inactivated FCS, 2 mM L-glutamine, 200 IU/ml penicillin, 100 µg/ml streptomycin and 1× non-essential amino acids (all from Biochrom, Berlin, Germany).

### Priming of Monocytes

For priming of isolated monocytes, cells were harvested after resting for 40 hours at 37°C and 7% CO_2,_ washed once with complete McCoy’s 5a medium and resuspended (1×10^6^ cells/ml). The resting period was chosen to minimize potential effects of cell activation during isolation procedure. Cells were then stimulated with *E. coli* 055:B5 derived LPS for 2 hours (10 ng/ml, Sigma-Aldrich, Taufkirchen, Germany).

### Adhesion, Migration and Chemotaxis

For determination of cell adhesion, 96-well flat-bottom plastic tissue-culture plates were treated with human fibronectin (50 µg/ml, BD Biosciences, Heidelberg, Germany) for 1 hour at 37°C. Monocytes (2×10^5^) were seeded in triplicates and incubated at 37°C and 7% CO_2_ for 4 hours. Non-adhering cells were removed by washing twice; remaining adherent cells were fixed with 2% glutaraldehyde (Sigma-Aldrich, Taufkirchen, Germany) for 10 min. Wells were washed two times with H_2_O and subsequently stained with 0.5% crystal violet (Merck, Darmstadt, Germany) in 2% EtOH (pH 6.0) for an additional 15 min at room temperature. Finally, wells were washed three times and lysed. 10% acetic acid was added and staining was quantified measuring the OD at 560 nm using an Asys Expert 96 Microplate ELISA reader (Anthos Mikrosysteme, Krefeld, Germany).

Monocyte migration and chemotaxis assays were performed in transwell plates (5 µm pore size) using Leukotriene B4 (LTB4, 100 nM, Cayman Chemical, Ann Arbor, MI, USA) as an additional chemoattractant. The modified Boyden chamber assay was used according to the manufacturer’s instructions (Corning, Lowell, MA, USA). Monocytes (2.5×10^5^) were seeded on top of the membrane and cell numbers in the lower chamber were quantified by counting after 4 hours of incubation at 37°C and 7% CO_2_. All analyses were performed in triplicate.

### *Escherichia coli* Phagocytosis

Cultured cells were harvested and resuspended (1×10^6^ cells/ml) in complete McCoy’s 5a medium together with 10 MOI fluorescein labeled *E. coli* supplemented with 15 µl human AB-serum and mixed thoroughly. The cells were incubated with *E. coli* at 37°C for 30 minutes. After incubation cells were washed two times with ice-cold McCoy’s 5a medium and analyzed by flow cytometry as described above. Phagocytic internalization of *E. coli* was confirmed by fluorescence microscopy.

### Oxidative Burst

Monocytes were stimulated for 2 hours at 37°C with *E. coli* 055:B5 derived LPS (10 ng/ml, Sigma-Aldrich, Taufkirchen, Germany) in the presence of 15 µM Dihydrorhodamine 123 (DHR, Merck, Darmstadt, Germany) for the final 15 min. Monocytes were placed on ice after incubation and analyzed by flow cytometry as described above.

### Analysis of Cytokine and Chemokine Levels

Cytokine and chemokine secretion of monocytes before and after LPS treatment was measured in cell supernatants. Human TNFα, IL1β, IL6, IL10, CCL2, CCL5, CXCL9, and CXCL10 were measured by Cytometric Bead Array (CBA, human chemokine kit and human inflammatory cytokine kit, BD Bioscience, Heidelberg, Germany) according to the manufacturer’s instructions and subsequently analyzed by flow cytometry. Human IL8 was measured using a commercial enzyme-linked immunosorbent assays (ELISA) according to the manufacturer’s instructions (BD Biosciences, Heidelberg, Germany).

### Quantitative Real-time PCR

Complementary DNA was synthesized from 1 µg of total RNA using SuperScript II RNase H-reverse transcriptase (Invitrogen, Carlsbad, CA, USA) with oligo_18_ dT primers. Primers used for RT-PCR analysis are given in Table 2. Real-time RT-PCR was performed by using an ABI PRISM 7900 (Applied Biosystems, Foster City, CA, USA) with a KAPA SYBR® FAST qPCR Kit (Kapa Biosystems, Woburn, MA, USA) as described previously [10]. Gene expression was normalized to the endogenous housekeeping control gene ribosomal protein L13a (RPL) and relative expression of respective genes was calculated by the comparative threshold cycle method. All analyses were performed in duplicate.

**Table 2:** Primer sequences for RT-PCR.

Gene	Forward Primer Sequence (5′-3′)	Reverse Primer Sequence (5′-3′)
TNFα	CTT CTC GAA CCC CGA GTG AC	TGA GGT ACA GGC CCT CTG ATG
IL1β	GCG GCC AGG ATA TAA CTG ACT TC	TCC ACA TTC AGC ACA GGA CTC TC
IL6	AGA GGC ACT GGC AGA AAA CAA C	AGG CAA GTC TCC TCA TTG AAT CC
IL8	CTT GTT CCA CTG TGC CTT GGT T	GCT TCC ACA TGT CCT CAC AAC AT
IL10	GCT GAG AAC CAA GAC CCA GAC A	CGG CCT TGC TCT TGT TTT CA
CCL2	TCG CCT CCA GCA TGA AAG TC	TTG CAT CTG GCT GAG CGA G
CCL5	CAG TGG CAA GTG CTC CAA CC	CCA TCC TAG CTC ATC TCC AAA GAG T
CXCL9	GAC CTT AAA CAA TTT GCC CCA AG	TCC TTC ACA TCT GCT GAA TCT GG
CXCL10	GCA AGC CAA TTT TGT CCA CG	ACA TTT CCT TGC TAA CTG CTT TCA G

### Statistics

Data are expressed as mean ± standard error of the mean (SEM) except when stated otherwise and were assessed using the Student`s t-test. P values less than 0.05 were considered to be statistically significant. All calculations were performed using SPSS version 14 (SPSS Inc, Chicago, IL, USA).

## Results

### Expression of Cell Surface Molecules

**Figure 1:**
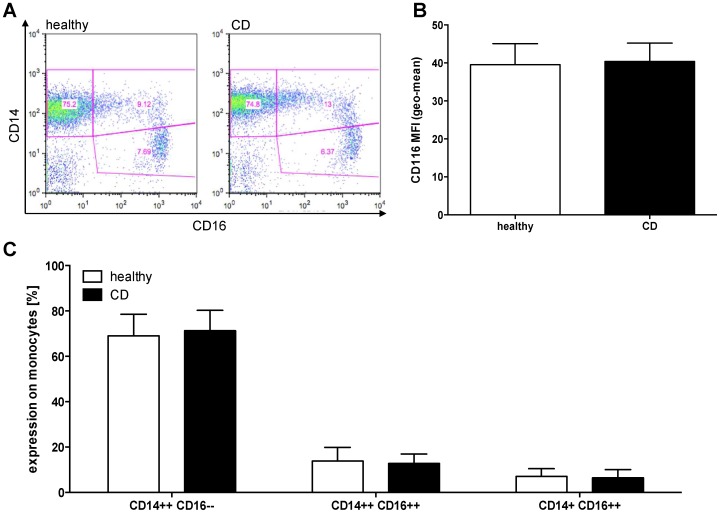
Cell surface molecule expression. (A) Example of CD14 and CD16 expression on naïve PBMCs after Ficoll isolation gated for viable monocytes. Upper left quadrant shows CD14++/CD16– monocytes, upper right quadrant shows CD14++/CD16++ monocytes, and CD14+/CD16++ monocytes are represented in the lower right quadrant. (B) Shown is the CD116 expression on naïve monocytes of healthy controls (n = 11) and patients with CD in remission (n = 12) as mean fluorescence intensity. (C) Cell surface expression of CD14 and CD16 on viable PBMC monocytes of healthy controls (n = 17) and patients with CD (n = 18) analyzed by flow cytometry as shown in (A). All bars represent means ± SEM.

We investigated whether differences exist in the CD14/CD16 subsets of monocytes from patients with quiescent CD as described for patients with active disease [11]. However, we found that there is no difference among these subsets (Figure 1A and C). The CD14++/CD16– subset represented 69.1% ±2.3% of monocytes of healthy controls (HC) and 71.3% ±2.1% of monocytes of included patients with CD, respectively. In addition, we found no significant differences in the CD14++/CD16++ and the CD14+/CD16++ subsets of HC and CD patients. Furthermore, we analyzed the expression of the GM-CSF-receptor (CD116) as there are reports of defective leukocyte CD116 expression and function in IBD [12]. In our analyses CD116 expression did not differ between monocytes derived from HC (MFI 39.5±5.6) and CD (MFI 40.3±4.9).

### Monocyte Functions

**Figure 2:**
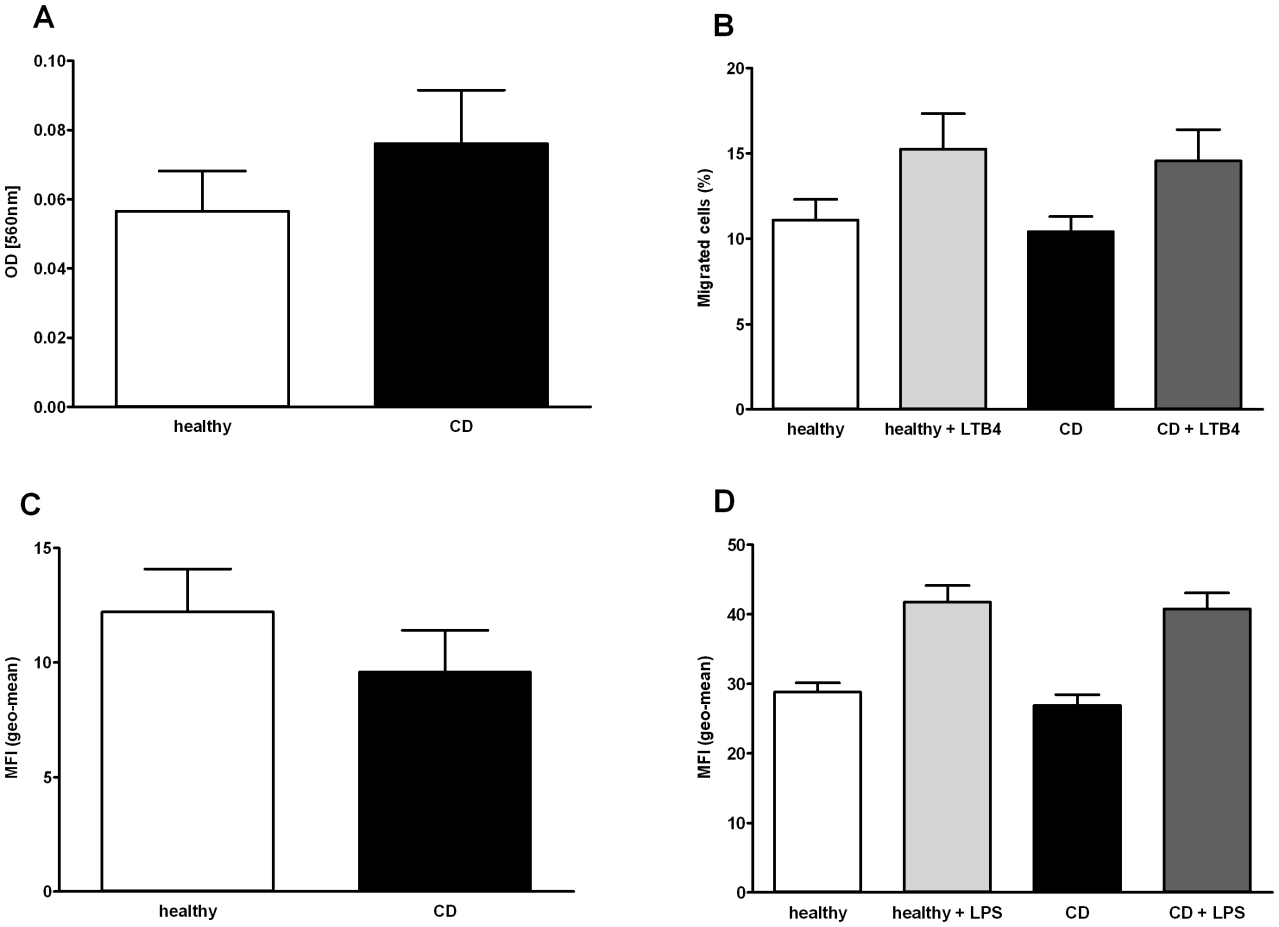
Functional cell assays. (A) Adhesion of monocytes of healthy controls (n = 15) and CD (n = 16) to fibronectin-coated plastic surface. (B) Migration and chemotaxis studies of monocytes of healthy controls (n = 5) and patients with CD (n = 5) using a modified Boyden chamber and LTB4 as a chemoattractant. (C) Phagocytosis of FITC-labeled *E. coli* by monocytes of healthy controls (n = 12) and patients with CD (n = 11). (D) Production of reactive oxygen species (ROS) by monocytes of healthy controls (n = 11) and patients with CD (n = 12) with and without further LPS stimulation for 2 hours. All bars represent means ± SEM.

To determine whether CD monocytes are functionally impaired, we performed several functional assays. We found that CD monocytes have no impairment in adhesion (OD [560 nm] HC 0.057% ±0.012% vs. CD 0.076% ±0.015%), migration (HC 11.1% ±1.2% vs. CD 10.4% ±0.9%) and chemotaxis towards LTB4 (HC 15.3% ±2.1% vs. CD 14.6% ±1.8%) (Figure 2A and B).

Phagocytosis of bacteria is a fundamental capacity of monocytes. CD monocytes showed similar phagocytosis capabilities in the presence of FITC-labeled *Escherichia coli (E. coli)* (MFI 9.6±1.8) compared with monocytes of HC (MFI 12.2±1.9) (Figure 2C).

We found no differences in the production of reactive oxygen species (ROS) by monocytes from patients with quiescent CD compared to healthy controls, neither in the presence of LPS (MFI HC 41.7±2.3 vs. CD 40.8±2.3) nor without LPS (MFI HC 28.8±1.3 vs. CD 26.9±1.5).

### Chemokine and Cytokine Expression

**Figure 3:**
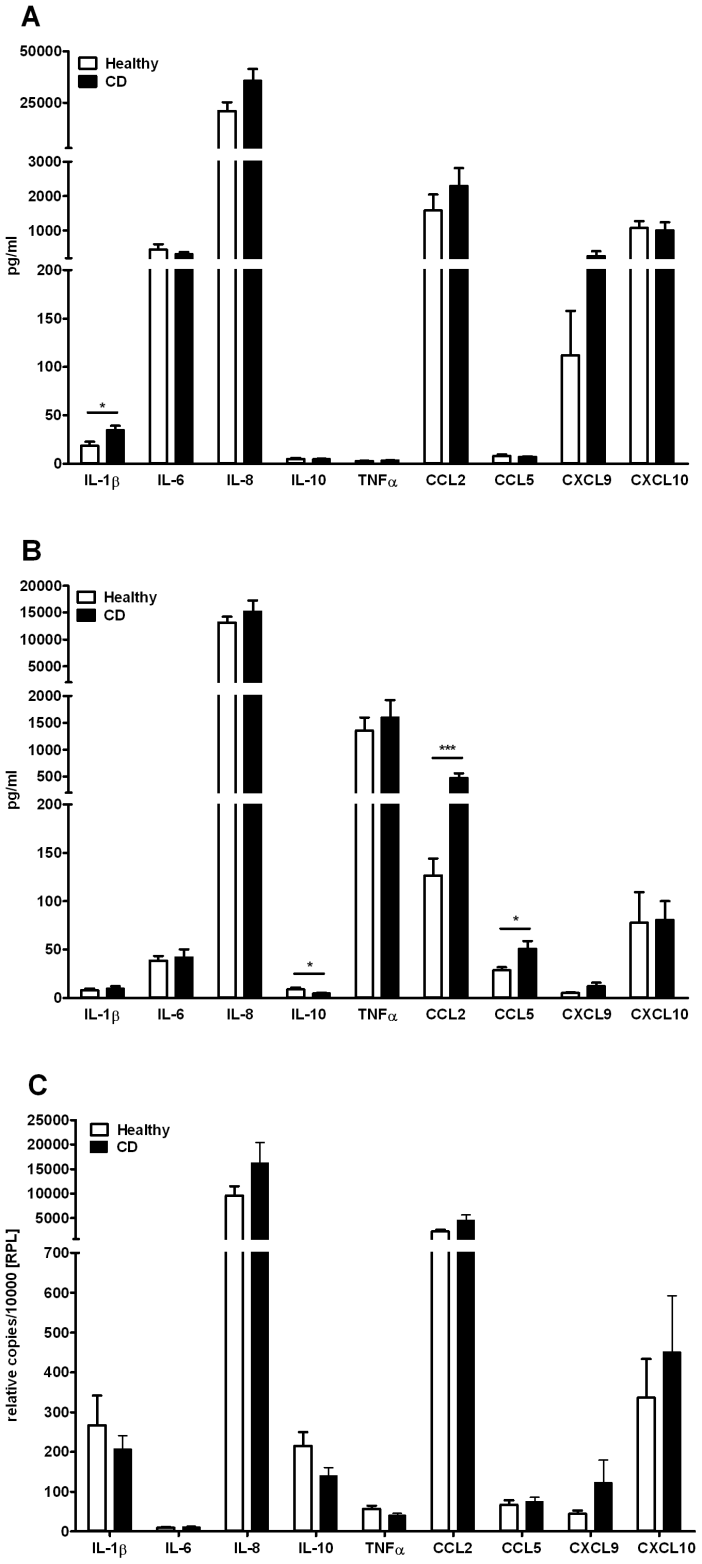
Cytokine expression and secretion. (A) Cytokine and chemokine secretion by resting monocytes of healthy controls (n = 19) and patients with CD in remission (n = 17). (B) Cytokine and chemokine secretion by monocytes of healthy controls (n = 13) and patients with CD (n = 13) stimulated with LPS for 2 hours. (C) mRNA expression levels of resting monocytes of healthy controls (n = 13) and patients with CD (n = 14). Error bars indicate SEM. *, P<0.05; ***, P<0.001.

We analyzed cytokine and chemokine secretion of freshly isolated monocytes after resting for 40 hours. Monocytes of CD patients in remission showed significantly higher levels of IL1β (HC 18.2 pg/ml ±4.3 pg/ml vs. CD 34.7 pg/ml ±4.5 pg/ml; P<0.05). There was no significant difference in the levels of TNFα, IL6, IL8, IL10, CCL2, CCL5, CXCL9, and CXCL10 (Figure 3A).

In addition, peripheral blood monocytes from patients with CD in remission showed a significantly higher secretion of the chemokines CCL2 (HC 126.8 pg/ml ±17.4 pg/ml vs. CD 472.8 pg/ml ±89.3 pg/ml; P<0.001) and CCL5 (HC 28.4 pg/ml ±3.2 pg/ml vs. CD 51.0±7.6 pg/ml; P<0.05) after priming with LPS. In contrast, the levels of anti-inflammatory IL10 were significantly lower in monocytes of included CD patients (HC 8.8 pg/ml ±1.9 pg/ml vs. CD 4.5 pg/ml ±0.9 pg/ml; P<0.05). Production of TNFα, IL1β, IL6, IL8, CXCL9, and CXCL10 was not different between monocytes from healthy controls compared to CD patients (Figure 3B).

Quantitative real-time PCR analysis also showed that there were no differences in gene expression of IL1β, IL6, IL8, IL10, TNFα, CCL2, CCL5, CXCL9, and CXCL10 in naïve monocytes (Figure 3C).

## Discussion

It was realized more than 30 years ago that the acute inflammatory response and neutrophil recruitment is impaired in CD [13]. Recent studies on the pathogenesis of inflammatory bowel diseases (IBD) further support the immunodeficiency model of Crohn’s disease. The focus has shifted from the primary defect in adaptive immunity to deficient acute inflammation in the intestinal mucosa with diminished macrophage cytokine production and neutrophil recruitment leading to a reduced bacterial clearance. Given that blood monocytes are the exclusive source of macrophages in the inflamed intestinal mucosa, here we have attempted to characterize peripheral blood monocyte populations in CD patients. The aim was to explore whether functional changes in resulting tissue macrophages are already detectable in blood monocytes. Our results suggest, that peripheral blood monocytes of CD patients in clinical remission are phenotypically and functionally indistinguishable from those of healthy controls.

Previous studies investigating functions of peripheral blood monocytes in CD have reported conflicting results. Mee *et al.* showed that peripheral blood monocyte counts, phagocytosis (*Staphylococcus aureus*), and intracellular killing are not impaired in CD. They concluded that peripheral blood monocytes in CD patients are rather activated and that granulomata in CD are unlikely to result from a defect in the microbicidal functions of monocytes [14], [15]. A study on the expression of cell surface molecules showed an increased MFI of surface molecules CD86/B7-2, CD18, and ICAM-1 on peripheral blood monocytes from patients with CD, which also indicates that monocytes from CD patients are activated and may have an increased antigen-presenting function by providing increased costimulatory signals (e.g. CD86/B7-2) [16]. Okabe *et al.* observed a reduced immunologic activity in CD monocytes [17], an increased superoxide production in peripheral blood monocytes of CD patients (determined by chemiluminescence) [18] as well as diminished phagocytic activity (determined by automated laser flow cytometry) [19]. On the other hand, Whorwell *et al.* reported that phagocytosis (*Candida albicans*) and undirected motility are significantly increased in blood monocytes of CD patients when compared with controls. In addition, they found no difference in the chemotaxis (towards zymosan-activated serum) between the disease and control group [20]. Baldessano *et al.* showed that freshly isolated monocytes from patients with inactive CD release equivalent amounts of superoxide anion (measured by superoxide dismutase inhibitable reduction of ferricytochrome c) when compared with monocytes from healthy controls. However, freshly isolated monocytes from patients with active CD showed a significantly enhanced respiratory burst and responded in a similar fashion to LPS-primed monocytes [21]. Miura *et al.* investigated the phagocytosis (yeast particles), monocyte polykaryon formation and accessory cell function of monocyte/macrophages in CD patients. They postulated that peripheral blood monocytes from patients with CD have abnormal functions, which may be involved in the pathogenesis of the granuloma and giant cell formation in CD patients [22].

Differences in the methodology used and the heterogeneity of the included study patients in previous studies may have led to the divergent data. Thus, we systematically studied phenotypic and functional characteristics of peripheral blood monocytes of patients with definitive but inactive CD compared with healthy controls. In order to specifically characterize potential intrinsic alterations of monocyte subpopulations in CD patients and to exclude potential *in vivo* effects of immunosuppressive and immunomodulatory medications, we exclusively included CD patients in clinical remission. This approach allowed comparison of our results with findings from a recent study by Smith *et al.* that utilized monocyte-derived macrophages from quiescent CD patients to identify a defective mechanism [5]. In the present study we report that adherence to plastic surfaces, spontaneous migration, chemotaxis towards Leukotriene B4, phagocytosis of *E. coli*, and production of reactive oxygen species is not impaired in peripheral blood monocytes of patients with inactive CD. LTB4 was chosen for our chemotaxis studies because of its ability to induce the adhesion and activation of leukocytes on the endothelium, allowing their extravasation into (intestinal) tissue. *E. coli* was chosen for the phagocytosis studies because of its abundance in intestinal flora. Our results show that cell functions of peripheral blood monocytes of CD patients are not intrinsically defective *per se*. However, the immunological activity of monocytes may potentially differ in the context of active disease, in the milieu of the inflamed intestinal mucosa, or after further cell differentiation/maturation. Hence, Smith *et al.* have revealed deficient secretion of the (pro-) inflammatory cytokines TNFα, IL4, IL6, IL5, IL13, IL15, IL12, IL17, IFNγ, and IL1β by macrophages from patients with inactive CD following exposure to *E. coli* [5]. In the present study we were able to demonstrate that the expression and release of TNFα, IFNγ, IL4, IL6, IL8, IL13, IL17, CXCL9, and CXCL10 from peripheral blood monocytes is not altered in patients with inactive CD compared to healthy controls. Furthermore, we observed that peripheral blood monocytes of CD patients secreted significant higher levels of pro-inflammatory IL1β and, upon LPS priming, significantly lower levels of anti-inflammatory IL10 compared to those of healthy controls. Interestingly, Smith *et al.* found no difference in the secretion of IL1β and IL10 by macrophages from CD patients in response to *E. coli* [5]. However, we cannot exclude that some of the patients were not in complete remission, even though the majority of the included patients with CD had a HBI score <3, and were thus very likely in remission according to the Crohn’s disease activity index (CDAI) [9]. However, clinical disease activity scores cannot sufficiently reflect subclinical intestinal inflammation and might be hindered by inaccuracy as a result of subjective components.

A subset of blood monocytes expressing LPS co-receptor CD14 and the low-affinity FCγ receptor CD16 (CD14+CD16+) has been identified previously as a major proinflammatory cell population characterized by low production of IL10 and high levels of IL1β, TNFα and IL12 [23]. CD14+CD16+ peripheral blood monocytes are increased in active CD and CD14+CD16+ cells are a major contributor to the inflammatory infiltrate in CD mucosa [24], [25]. Thus, our findings further support the assumption that peripheral blood monocytes from CD patients with quiescent disease are immunological competent rather than functionally impaired. CD14+CD16+ peripheral blood monocytes showed normal levels and were not increased in our CD cohort compared to healthy controls, possibly due the fact that the patients were in clinical remission. Furthermore, it has been reported that the expression of circulating granulocyte-macrophage colony-stimulating factor receptor (CD116) on IBD monocytes from IBD patients is decreased compared to healthy controls (independent of disease activity) [12]. However, we did not observe lower CD116 expression levels on peripheral blood monocytes of patients with quiescent CD, which could be explained by the fact that the IBD-associated CD116 repression is more prominent in patients with ulcerative colitis compared to Crohn’s disease [12].

In addition, we found a significantly enhanced release of the chemokines CCL2 (MCP-1) and CCL5 (RANTES) of LPS-primed peripheral blood monocytes of patients with quiescent CD. Both chemokines play an active role in the recruitment of leukocytes to the sites of inflammation. The elevated secretion of chemokines by peripheral blood monocytes of patients with inactive CD provides further evidence for our observation that the monocyte compartment is not functionally impaired in quiescent CD but instead represents immunocompetent cells having the potential to initiate an effective immune response.

Despite the relatively small number of included CD patients, our data were highly consistent within each group of subjects. Nevertheless, further studies may elucidate the impact of the disease activity on monocyte functions in CD patients. Furthermore, comparative studies in peripheral blood monocytes as well as tissue macrophages in patients with active CD patients may further illuminate the underlying immune mechanisms. In addition, other stimulants of Toll-like receptors (TLRs), as well as nucleotide oligomerization domains (NODs), should be investigated, and CD patients should be characterized for their genetic carriage of innate immune gene polymorphisms.

In conclusion, based on our phenotypic and functional studies, peripheral blood monocytes from CD patients in clinical remission were not impaired compared to healthy controls. Our results highlight that defective innate immune mechanisms in CD seems to play a role in the (inflamed) intestinal mucosa rather than in peripheral blood.
